# Proteases Involved in Leader Peptide Removal during RiPP Biosynthesis

**DOI:** 10.1021/acsbiomedchemau.3c00059

**Published:** 2023-12-13

**Authors:** Sara M. Eslami, Wilfred A. van der Donk

**Affiliations:** † Department of Chemistry, University of Illinois at Urbana−Champaign, Urbana, Illinois 61801, United States; ‡ Howard Hughes Medical Institute, University of Illinois at Urbana−Champaign, Urbana, Illinois 61801, United States

**Keywords:** protease, peptidase, RiPP, macrocyclase, metalloprotease, proteolysis, maturation, cyclic peptides, leader peptide

## Abstract

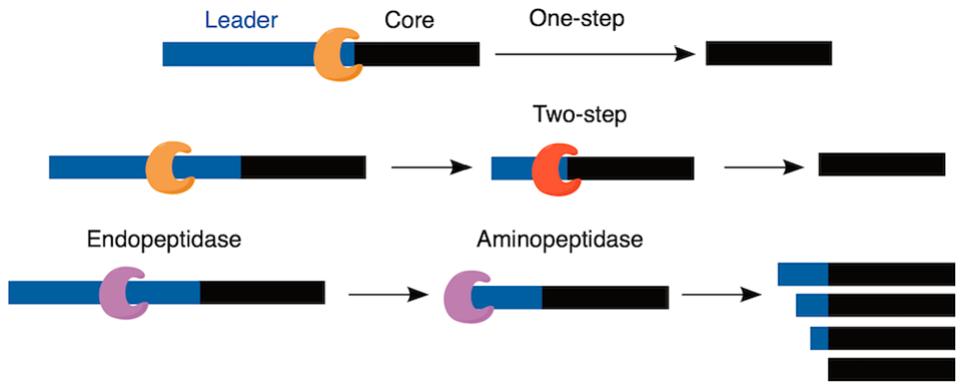

Ribosomally synthesized and post-translationally modified peptides (RiPPs) have received much attention in recent years because of their promising bioactivities and the portability of their biosynthetic pathways. Heterologous expression studies of RiPP biosynthetic enzymes identified by genome mining often leave a leader peptide on the final product to prevent toxicity to the host and to allow the attachment of a genetically encoded affinity purification tag. Removal of the leader peptide to produce the mature natural product is then carried out in vitro with either a commercial protease or a protease that fulfills this task in the producing organism. This review covers the advances in characterizing these latter cognate proteases from bacterial RiPPs and their utility as sequence-dependent proteases. The strategies employed for leader peptide removal have been shown to be remarkably diverse. They include one-step removal by a single protease, two-step removal by two dedicated proteases, and endoproteinase activity followed by aminopeptidase activity by the same protease. Similarly, the localization of the proteolytic step varies from cytoplasmic cleavage to leader peptide removal during secretion to extracellular leader peptide removal. Finally, substrate recognition ranges from highly sequence specific with respect to the leader and/or modified core peptide to nonsequence specific mechanisms.

## Introduction

**Figure 1:**
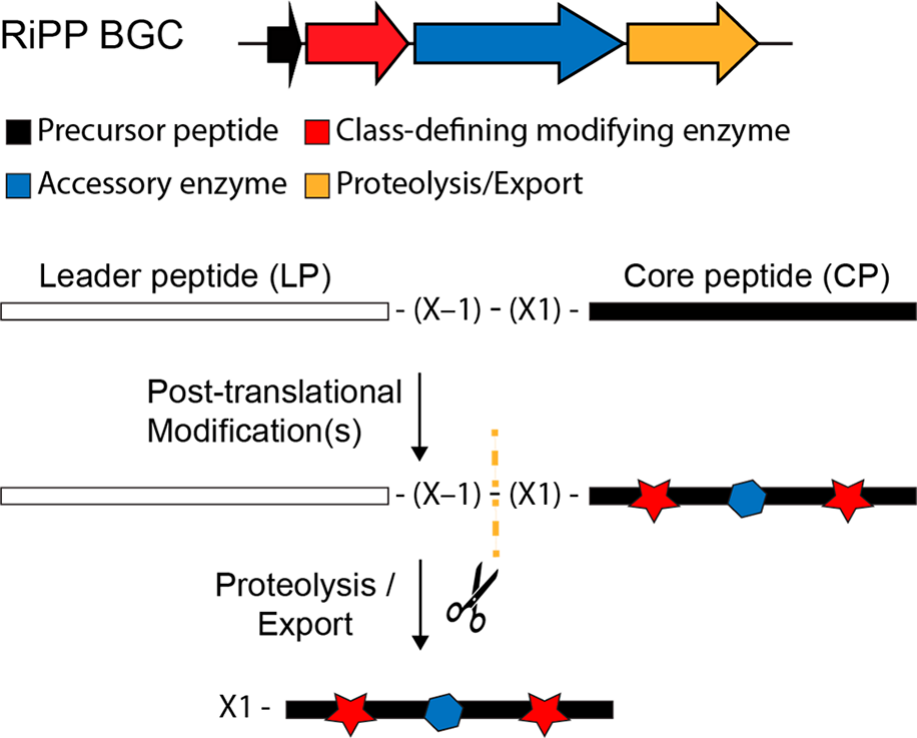
Generic overview of RiPP biosynthesis. A precursor peptide is modified by one or more class-defining modifying enzymes that introduce the PTM(s) that are characteristic for a specific RiPP class. In some cases, accessory enzymes may introduce additional non-class-defining tailoring PTMs. The BGC often, but not always, encodes export machinery and/or one or more proteases to remove the leader peptide (LP). In some examples the protease is fused to the export proteins, whereas in other cases these two functions are in separate polypeptides. In RiPP systems, the residues at the protease cleavage site are denoted X–1 and X1,^7^ which are the P1 and P1′ positions in protease nomenclature.^8^

Ribosomally synthesized and post-translationally modified peptides (RiPPs) comprise a subset of natural products found in all domains of life and harbor a multitude of bioactivities.^1−5^ RiPP biosynthetic gene clusters (BGCs) encode pathways that follow a common logic wherein a genetically encoded peptide is post-translationally modified by enzymes that are mostly encoded in the BGC.^1^ The precursor peptide is generally divided into a leader peptide (LP) region involved in enzyme recognition followed by the core peptide (CP) that undergoes post-translational modification (PTM) and is released via proteolytic cleavage (Figure 1).^6^

Most early investigations into RiPP natural products have been bioactivity-guided efforts leading to notable discoveries such as nisin, microcin B17, and darobactin.^9−13^ Advances in bioinformatic technologies, such as antiSMASH, BAGEL, RODEO, RiPPer, the tools of the Enzyme Function Initiative, and many others, have enabled rapid identification of RiPP BGCs within the ever-growing genome databases.^1,14−22^ Consequently, renewed efforts have focused on the discovery of novel RiPPs by genome mining (e.g., refs (1) and (23−35)). In addition, many studies have reported engineering of RiPP pathways to make new-to-nature structures and hybrid RiPPs.^1,36−39^ A current bottleneck in such research is the identification of proteases that recognize the cognate RiPPs and remove the LP to yield the final, mature compound. While many studies have made use of commercially available enzymes such as the endoproteinases trypsin, GluC, LysC, AspN, Tobacco Etch Virus protease, and Factor Xa, their use requires the introduction of non-native amino acids at the end of the LP, which may not always be tolerated by the biosynthetic enzymes. Alternatively, making use of existing cleavage sites for these proteases typically leads to compounds with residues remaining from the LP. In recent years, significant progress has been made toward the identification and use of the cognate RiPP proteases or protease domains, providing access to the native compounds.

Herein, we summarize the current state of identifying and utilizing dedicated leader-peptide-removing proteases from bacterial RiPP pathways, identifying gaps in our knowledge, as well as areas for future research. We organized the review by protease class and MEROPS database classification^40^ when available rather than RiPP family. Table 1 connects proteases to RiPP families, demonstrating that sometimes multiple protease types are used within a certain RiPP family. As will be discussed, quite a few RiPPs are matured by two independent successive proteolytic events for reasons that are not well understood.^41−48^ In Table 1, and throughout the review, we use the established nomenclature for RiPP precursor peptides, in which residues in the CP are numbered with positive numbers counting up from the protease cleavage site and residues in the LP are indicated with negative numbers counting down (i.e., more negative) from the cleavage site toward the N-terminus (Figure 1).^7^ The scope of the current review does not include proteases involved in the biosynthesis of eukaryotic RiPPs, and we refer the reader to other publications regarding research on these systems.^49−60^ This perspective is also not focused on the molecular mechanisms of these proteases, but we do discuss substrate specificity when known.

**Table 1:**
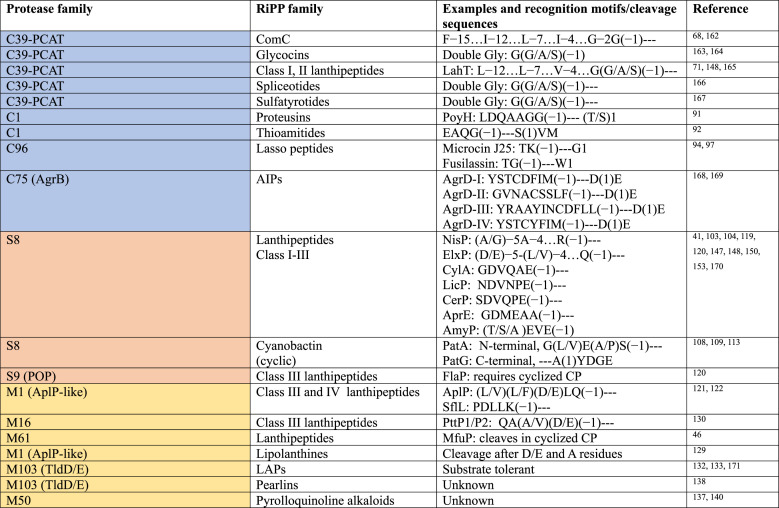

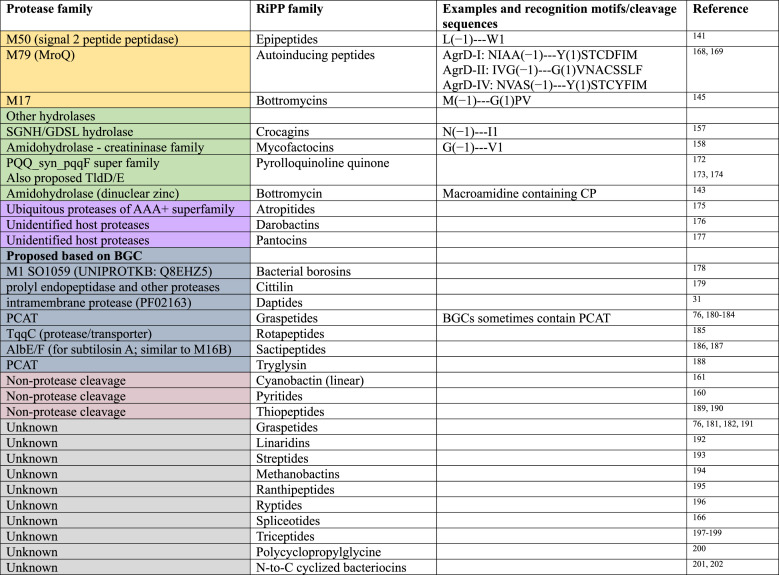
Proteases Involved in Leader Peptide Removal during RiPP Biosynthesis Organized by Protease Family^a^

a References (31, 41, 46, 68, 71, 76, 91, 92, 94, 97, 103, 104, 108, 109, 113, 119−122, 130, 132, 133, 137, 138, 140, 141, 143, 145, 147, 148, 150, 153, 157, 158, and 160−202).

## Cysteine Family Proteases

Within this section, we review the involvement of cysteine family proteases in the maturation of lanthipeptides (C39), polytheonamides (C1A), and lasso peptides (C96). A description of the mechanism used by ubiquitous bifunctional peptidase-containing ATP-binding transporters (PCATs) is provided, as well as the minimal LP recognition motifs for each RiPP–cysteine protease pair when known.

Several RiPP classes harbor cysteine family proteases that cleave after a canonical double glycine-like motif (GG, GA, or GS)^61,62^ at the end of the LP. Of the cysteine protease family, the most common type within RiPP BGCs is the PCATs, also known as ATP-binding cassette (ABC) transporter maturation and secretion (AMS) proteins.^61,62^ PCATs are characterized by the presence of an N-terminal papain-type C39 protease domain followed by an ABC transporter. These enzymes remove the LP at the double Gly motif, present in some of the most common RiPP precursor peptide families that resemble proteins in nitrogen fixation (NIF11 LPs) and nitrile hydratase (proteusin LPs). In addition to these very long LPs (>70 residues) that are used in a variety of different RiPP families especially in cyanobacteria (e.g., polytheonamides, thiazole/oxazole-containing peptides, and class II lanthipeptides),^63−65^ shorter versions ending in a double glycine motif and associated with PCATs are found in a wide variety of bacteria including Bacillota (Firmicutes), Enterobacterales, and Bacteriodales.^30,61,66,67^

**Figure 2:**
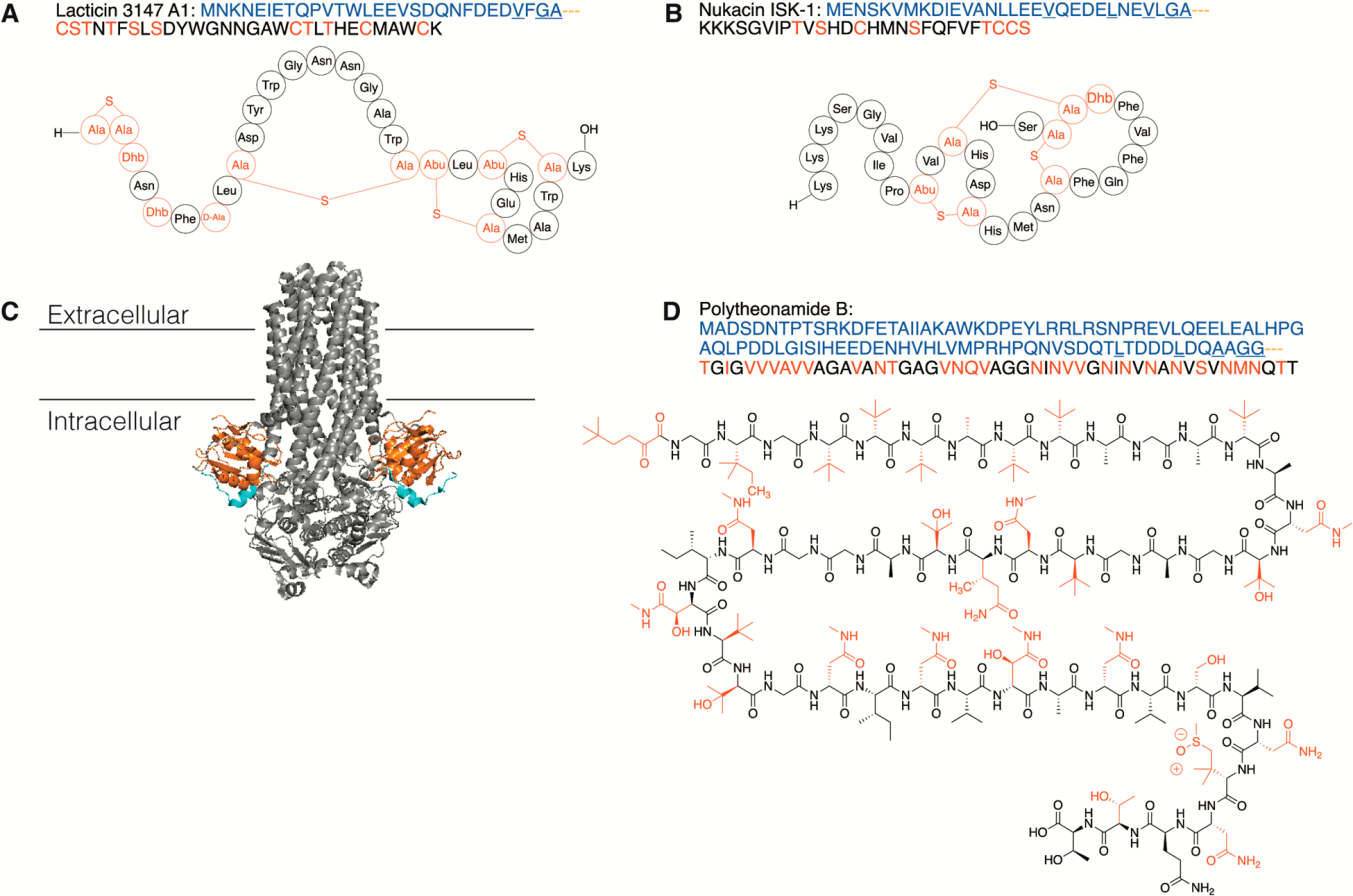
Representative examples of the cleavage of LPs by Cys proteases. (A) The lanthipeptide lacticin 3147 α, (B) the lanthipeptide nukacin ISK-1, (C) cryo-EM structure of PCAT1 (gray) with PEP domains (orange) bound to the peptide CtA (blue) (PDB 6V9Z), and (D) the proteusin polytheonamide B. The leader peptides are shown in blue, and the modified residues in the core peptides are shown in red. The double Gly-like motifs as well as the hydrophobic residues in positions −4, – 7, and −12 are underlined. In panels A and B, Ala-S-Ala denotes lanthionine cross-links and Abu-S-Ala denotes methyllanthionine cross-links.

An initial LP recognition sequence Phe(−15)-X_2_-Ile(−12)-X_3_-Leu(−7)-X_2_-Ile(−4) (Table 1) was identified for the PCAT ComA; ComA recognizes an amphipathic α-helical portion of the N-terminus of its substrate peptide and subsequently cleaves after a double glycine site to produce the mature ComC involved in the *Streptococcus pneumoniae* quorum sensing pathway.^68−70^ Providing a molecular explanation for this minimum recognition motif, an α-helical region was observed within the LP of the class II lanthipeptide precursor peptide LahA in a cocrystal structure with the protease domain LahT147 (the N-terminal 147 amino acids of the PCAT LahT). In the cocrystal structure, hydrophobic residues in the −4, – 7, and −12 positions (typically Leu, Ile, Val, Met, or Ala) along the helix were recognized via hydrophobic pockets within the LahT enzyme.^71^ The LahT PCAT is encoded in a BGC that contains as many as nine putative precursor peptides with conserved leader peptides but very diverse core peptides,^72^ explaining why it is able to remove the LP from many noncognate substrates.^71^ Indeed, LahT147 (and LahT150) has become a useful tool for removing LPs for many different RiPPs from a wide variety of families.^24,28,73−82^ Another interesting approach has been the covalent attachment of the C39 protease domain of BovT to the lanthipeptide synthetase BovM to generate the class II lanthipeptide bovicin HJ50.^83^ The excised LahT150 protease domain and the full length transporter LtnT involved in lacticin 3147 biosynthesis (Figure 2A) are quite tolerant in terms of both LP and CP residues as well as the form of the CP (modified or unmodified) as long as the recognition motif is present in the LP. However, other PCATs such as NukT (biosynthesis of the lanthipeptide nukacin-ISK, Figure 2B) are selective for their post-translationally modified peptide,^84,85^ and specific interactions between the transported peptide and the transmembrane domain (TMD) of PCATs have been reported.^86^ This finding may explain why excision of a protease domain from a PCAT does not always provide an active enzyme in the absence of the TMD.^85,87^

PCATs typically consist of an N-terminal C39 peptidase domain (PEP), a C-terminal nucleotide-binding domain (NBD), and a TMD. Crystallization of a full length PCAT from *Clostridium thermocellum* (PCAT1) illustrates the conformation of the enzyme in both ATP- and non-ATP-bound forms.^87^ At present, the structure of the cognate post-translationally modified substrate for PCAT1 has not been determined, but it is presumed to be a RiPP system. PCAT1 forms a dimer and exhibits an α-helical barrel structure within the cell membrane (Figure 2C). The peptidase domain is weakly associated with the transporter domain. When bound to ATP, a conformational change in PCAT1 closes the transmembrane tunnel and releases the peptidase domain from its association. Activity assays demonstrated that in this state, proteolytic activity was reduced.^87^ The origin of this reduced activity in the detached state has been explained through NMR studies.^88^ Cryo-electron microscopy (cryo-EM) studies of PCAT1 with the peptide substrate CtA that is encoded nearby provided insight into the mechanism of binding, proteolysis, and transport (Figure 2C).^89^ First, while the PCAT1 dimer may bind two substrates, only one peptide is conformationally primed for cleavage. The C-terminal portion of the primed substrate is inserted into the transmembrane barrel while the PEP cleaves the N-terminal LP. After ATP binding, a conformational change reorients the tunnel toward the extracellular space; this releases the core peptide to the outside and allows for dissociation of the peptidase with subsequent release of the LP. Another substrate may bind to the free PEP, and the hydrolysis of ATP reorients the PCAT toward the intracellular space where it is ready for substrate docking. PCATs are used for the biosynthesis of a wide variety of RiPPs including lanthipeptides, glycocins, spliceotides, sulfatyrotides, and graspetides (Table 1), but their use is not universal within these RiPP families.

Although the C1A family is uncommon in bacteria,^90^ such proteases have been found within certain RiPP BGCs (Table 1). Unlike C39-based PCATs, C1A proteases contain only a protease domain, with no additional transporter domain. The PoyH C1A protease involved in the biosynthesis of the proteusin polytheonamide (Figure 2D) has been shown to be substrate tolerant.^91^ PoyH processed PoyA variants bearing different PTMs as well as precursor peptides from other RiPP classes such as lanthipeptides and thiopeptides containing the LP recognition sequence LDQAAGG at the −7 to −1 positions (Table 1). Whereas PoyH showed robust activity, the C1A protease ThoK, involved in the generation of the thioamitide thioholgamide, demonstrated slow catalytic processing of its cognate (semi)modified precursor peptide.^92^ Such slow processing may be indicative of either a protective feature of the BGC in preventing premature proteolysis before the PTMs have been completed or the need for a zymogen activation step.^93^

Lasso peptides are generated by unique cysteine family proteases defined by the presence of a transglutaminase-like domain (C96 family). Proteolytic activity of McjB was confirmed via in vitro reconstitution of microcin J25 biosynthesis.^94^ Furthermore, an investigation into the mechanism of fusilassin (also called fuscanodin) maturation demonstrated the requirement of a RiPP recognition element (RRE) encoded separately from or fused to the protease for complete proteolysis.^95−97^ Proteolytic removal of the LP and macrolactam formation of the new N-terminal amine with a side chain carboxylate of a Asp/Glu residue appear coupled in lasso peptide biosynthesis. Although cyclization of the linear core peptide has been reported, in vitro it is generally a less efficient substrate than the full length peptide.^94,96,98^ Considerable substrate tolerance has been demonstrated for the fusilassin system by using chimeric substrates composed of a cognate LP for the protease and cyclase but variant core peptides, including at the P1′ position (residue 1 of the core peptide).^95,99^ The peptidase activity is greatly increased by the presence of the RRE domain and covariance data and NMR spectroscopy studies were used to provide a model for the interaction of the two proteins with the LP, which also provided an explanation for an invariant Thr residue at position −2 in the LP of lasso peptides.^97^ A recent report also documented bifunctional protease/transporter proteins involved in lasso peptide formation.^100^

## Serine Family Proteases

This section describes the involvement of serine family proteases in the maturation of class I, III, and IV lanthipeptides (S8, S9, and POPs) and cyanobactins (S8) (Table 1). LP recognition motifs are described for nisin-like and epilancin-like class I lanthipeptide maturation, and dual proteolytic cleavage during biosynthesis of the N-to-C cyclized cyanobactin patellamide involving serine proteases is discussed.

**Figure 3:**
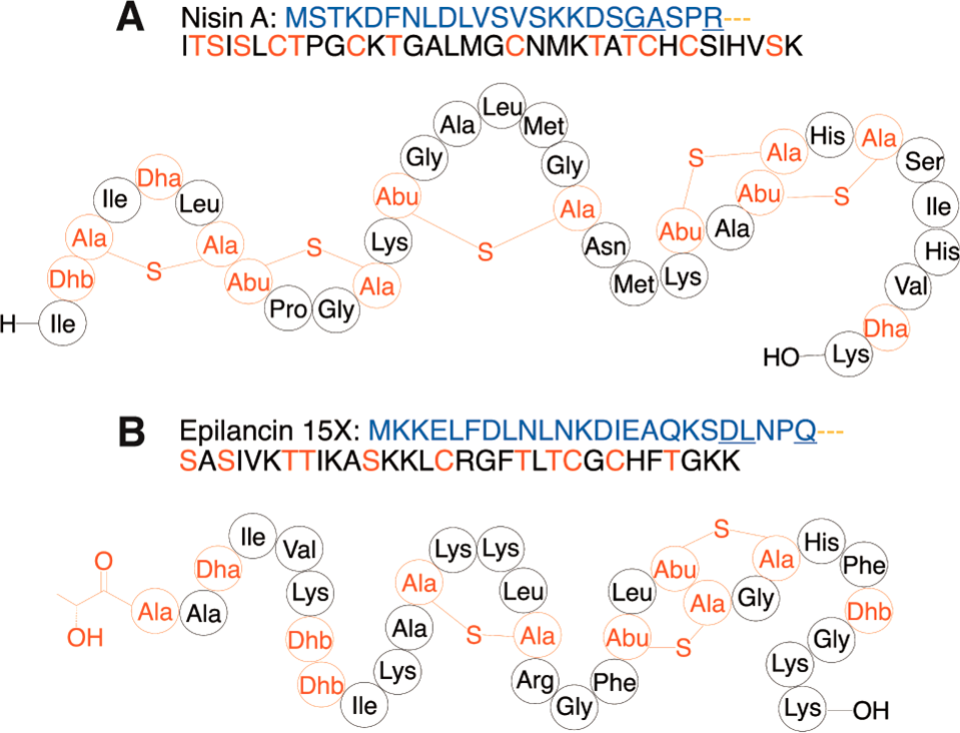
Representative examples of LP removal by serine proteases. (A) Nisin A and (B) epilancin 15x. The LP is shown in blue, and modifications in the CP are shown in red. Recognition sequences in the LP are underlined.

The biosynthesis of the class I lanthipeptide nisin has been thoroughly investigated and reviewed.^101^ The nisin protease NisP that removes the LP (Figure 3A) belongs to the subtilisin-like S8 family of serine proteases. NisP is translated as a prepro-protein before secretion, autoproteolysis, and extracellular peptidoglycan anchoring take place.^101^ The crystal structure of NisP revealed the autocatalytic cleavage of the C-terminal portion of the enzyme, although prevention of this cleavage did not inhibit NisP activity with NisA.^102^ Substrate recognition by NisP-like proteases of NisA-like peptides is dependent on the presence of a specific LP motif: (Ala/Gly–5)-(Ala–4)-X_2_-(Arg–1) (Table 1 and Figure 3A).^103^ The molecular details of how the residues in this motif are recognized by the enzyme are currently not available. Substrate specificity of NisP with respect to the core peptide appears to be less stringent, with proteolytic cleavage observed for NisA peptides that did not contain (methyl)lanthionine cross-links.^104^ However, the catalytic efficiency (*k*_cat_/*K*_m_) with unmodified NisA as the substrate was approximately 100-fold lower than with fully modified NisA. NisP was also active toward NisA substrates bearing varying numbers of (methyl)lanthionine rings. The higher efficiency with a fully modified substrate is mostly due to a higher *k*_cat_ value, suggesting that the rings are important for optimal orientation of the cleavage motif in the active site. The efficiency of NisP to remove the LP from nisin mutants in which the residue at the first position of the CP was changed to every other proteinogenic amino acid has also been investigated.^105^ These experiments showed that NisP was able to cleave the LP from all mutants except the I1P variant, although different efficiencies were observed.

The protease ElxP, involved in the biosynthesis of the class I lanthipeptide epilancin 15x, likewise belongs to the S8 family but recognizes an alternative motif, (Asp/Glu–5)-(Leu/Val–4)-X_2_-(Gln–1), in the LP of ElxA-like peptides (Figure 3B).^106,107^ Alanine substitutions of either Gln–1 or Leu–4 reduced the ElxP activity by an order of magnitude. Additionally, when the NisP recognition motif of NisA was replaced with that of ElxP, modified NisA was proteolyzed by the noncognate protease. Unlike NisP, ElxP is a cytoplasmic protease requiring tight control over the order of PTMs to prevent unproductive cleavage of the unmodified peptide. Like with NisP, the molecular details for substrate recognition are currently unresolved.

**Figure 4:**
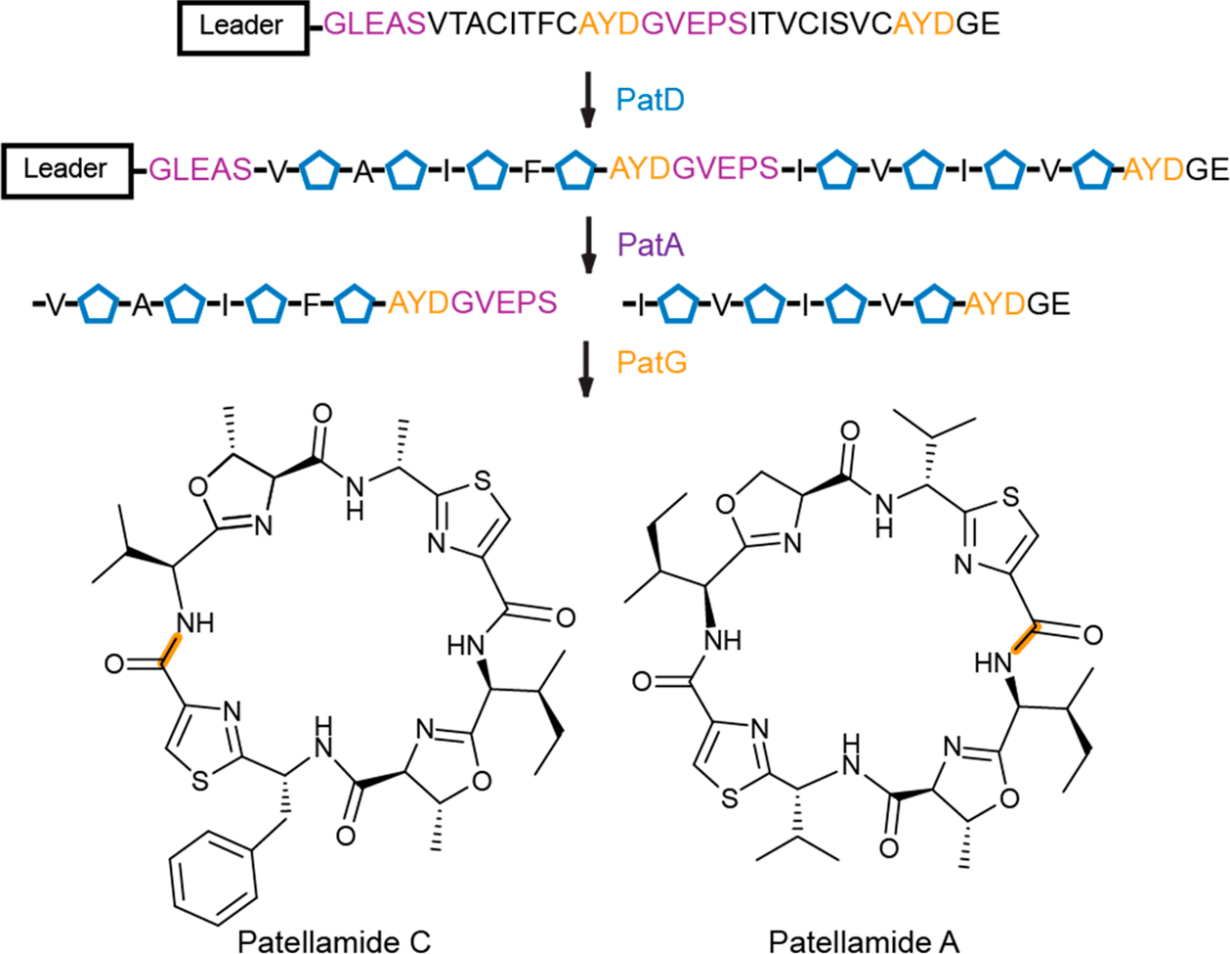
Biosynthetic processing of patellamides A and C. Pentagons represent thiazoles and oxazoles installed by PatD from Cys and Ser/Thr residues, respectively (blue). Cleavage by PatA (purple) and PatG (orange) are illustrated. PatG also catalyzes the N-to-C terminal cyclization, illustrated by the orange amide bonds.

The dual action of two subtilisin family proteases PatA and PatG is necessary for the formation of the circular cyanobactin patellamides A and C (Figure 4). While sharing over 40% similarity, PatA and PatG cleave at separate recognition sequences.^108,109^ During the biosynthesis of a large variety of cyclic cyanobactins,^110^ PatA-like enzymes first cleave after the consensus sequence G(L/V)E(A/P)S, followed by proteolysis by PatG-like enzymes before the AYDG sequence and subsequent macrocyclization (Figure 4). Given the unique proteolytic and macrocylization activities of PatG and its orthologues, these proteins are categorized as transamidating proteases distinct from other members of the subtilisin family.^109,111,112^ The cyanobactin macrocyclases have proven quite tolerant of the core peptide sequences and have therefore been used for engineering of a variety of cyclic peptides.^108,110,113−118^

S8 family proteases were also recently discovered in the processing of class III lanthipeptides. AmyP was identified in the production of gut-microbe derived peptides from *Bacillus amyloliquefaciens*; subsequent bioinformatic and mutational analyses illustrated a (T/S/A)EVE motif for protease activity (Table 1).^119^

Prolyl-oligopeptidases (POPs) belonging to the S9 serine protease family have been implicated in class III and IV lanthipeptide maturation.^120^ The protease FlaP, encoded in the BGC of the class III lanthipeptide flavipeptin, was selective for the fully modified FlaA peptide, only cleaving efficiently after the Pro(−12) residue in the LP when macrocycles were installed in the CP.^120^ At the time, it was not clear how residues −11 to −1 would be removed after FlaP cleavage. As discussed below, later studies suggested that metalloproteases that possess both endo- and aminopeptidase activity likely accomplish that task (vide infra).

## Metalloproteases

In this section, we provide an overview of the wide variety of metalloproteases involved in LP cleavage of class III and IV lanthipeptides (M1, M16, and M61), lipolanthines (M1), linear-azol(in)e-containing peptides (LAPs) (M103), pearlins (M103), and epipeptides (M50). The bifunctional endo- and exo-activities of the M1 proteases are discussed as well as the intriguing “pencil sharpener” mechanism proposed for M103 TldD/E proteases.

**Figure 5:**
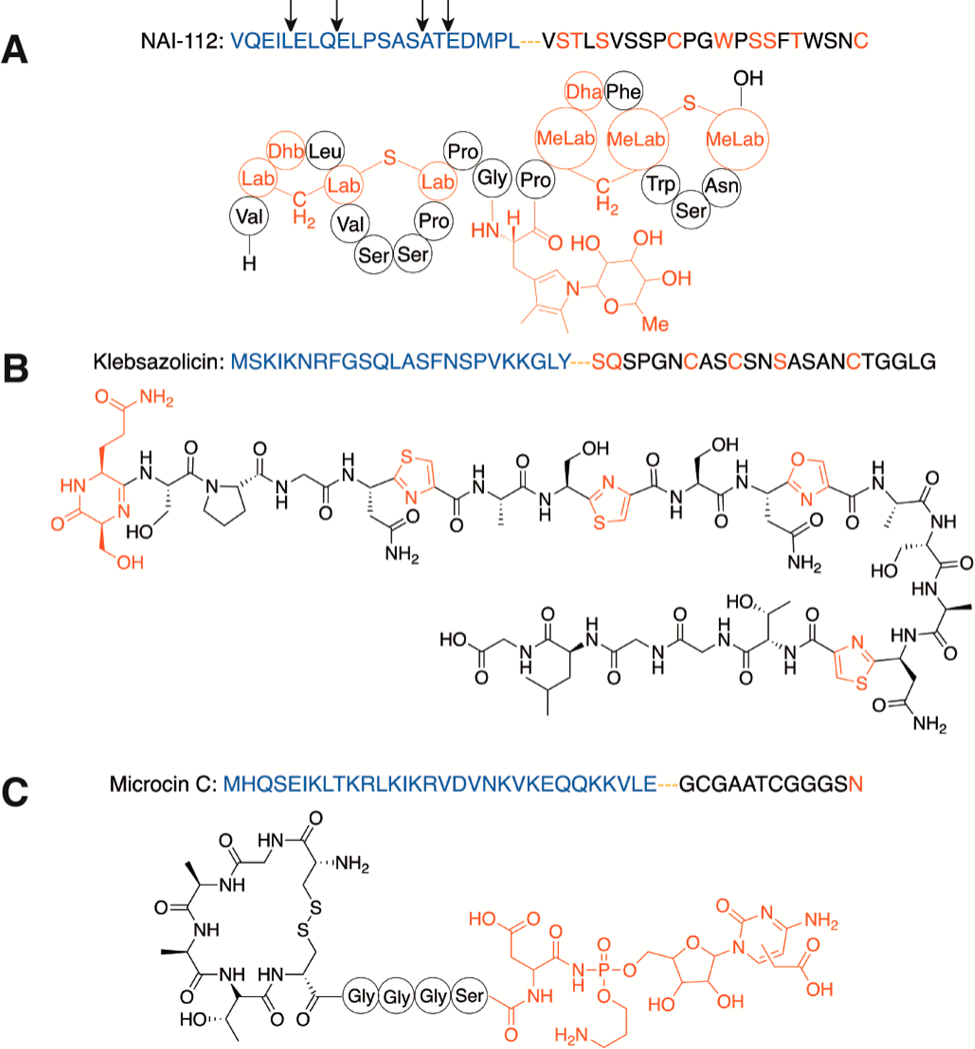
Representative examples of LP removal by metalloproteases. (A) NAI-112, (B) klebsazolicin, and (C) microcin C from *Y. pseudotuberculosis* (McC^Yps^). The LP is shown in blue, and the modification sites in the CP are shown in red. Lab = labionin; MeLab = methyllabionin. Black arrows indicate sites of AplP endopeptidase cleavage.

In 2019, AplP was identified as a bifunctional Zn-dependent protease from the M1 family that is necessary for the maturation of the class III lanthipeptide NAI-112 (Figure 5A).^121^ AplP displays dual activities, first cleaving the N-terminal LP after conserved E-(I/L)-(L/Q) and S-A-(S/T) sequence motifs as an endopeptidase (Table 1) followed by aminopeptidase trimming of the initial product to yield the final modified CP (Figure 5A).^121^ Unlike FlaP, AplP is active with both modified and unmodified precursor peptides. Whereas AplP involved in NAI-112 biosynthesis is encoded within the BGC, bioinformatic analyses revealed that genes for similar enzymes are present in the genomes of known class III and IV lanthipeptide producers but not in the biosynthetic gene loci.^121^ Indeed, AplP was demonstrated to cleave the LP of a series of different class III precursor peptides. More recently, an AplP-like enzyme (SflL) was also shown to be involved in the biosynthesis of the class IV lanthipeptide SflA. Although not encoded within the SflA BGC, the enzyme was active against SflA, recognizing the sequence PDLLK for initial endoproteinase activity after the Lys (Table 1), followed by further aminopeptidase trimming of the remainder of the LP.^122^ These findings with AplP and SflL explain why class III and IV lanthipeptides have been detected as a mixture of congeners when produced by the native organisms that differ in the number of amino acids that are removed by aminopeptidase activity.^123−128^

The biosynthetic pathway toward the lipolanthine microvionin also utilizes AplP-like Zn-dependent proteases termed MicP1 and MicP2 encoded within the genome of the native producer (outside of the BGC).^129^ MicP2 was uncharacteristically efficient compared to similar enzymes described, with cleavage observed after acidic amino acids and to a lesser extent the N-termini of Ala-Ala sequences.^129^ An α-helix-forming sequence (θxx)θxxθxxθ (θ = L, I, V, M, or T) in the LP was shown to be critical for PTM installation but not for MicP activity.

A recent study established a general workflow for identifying proteases that are encoded outside of RiPP BGCs.^130^ A correlation network was established between lanthipeptide precursor peptides and proteases from over 20,000 BGCs, with about one-third not containing a protease in the BGC. By first establishing correlations between precursor peptide sequences and proteases that are encoded within BGCs, predictions could be made for identifying proteases encoded outside of class III lanthipeptide BGCs. This workflow was first validated by the identification of the previously unknown S8 protease involved in the biosynthesis of the known class I lanthipeptide paenilan. The methodology was next extended to the discovery of Zn-dependent heteromeric proteases (M16) involved in the biosynthesis of new class III lanthipeptide families, the bacinapeptins and paenithopeptins.^130^ The protease complex termed PttP1/PttP2 displayed endopeptidase activity cleaving after a QAAD motif, followed by aminopeptidase activity. PttP1/PttP2 was not able to cleave the LP from an uncorrelated class III lanthipeptide; similarly, an uncorrelated AplP-like enzyme was inactive toward the precursor PttA1. These results illustrate the efficacy of correlational networking in identifying proteases for cognate precursor peptides, especially for proteases that are encoded outside of RiPP BGCs.

**Figure 6:**
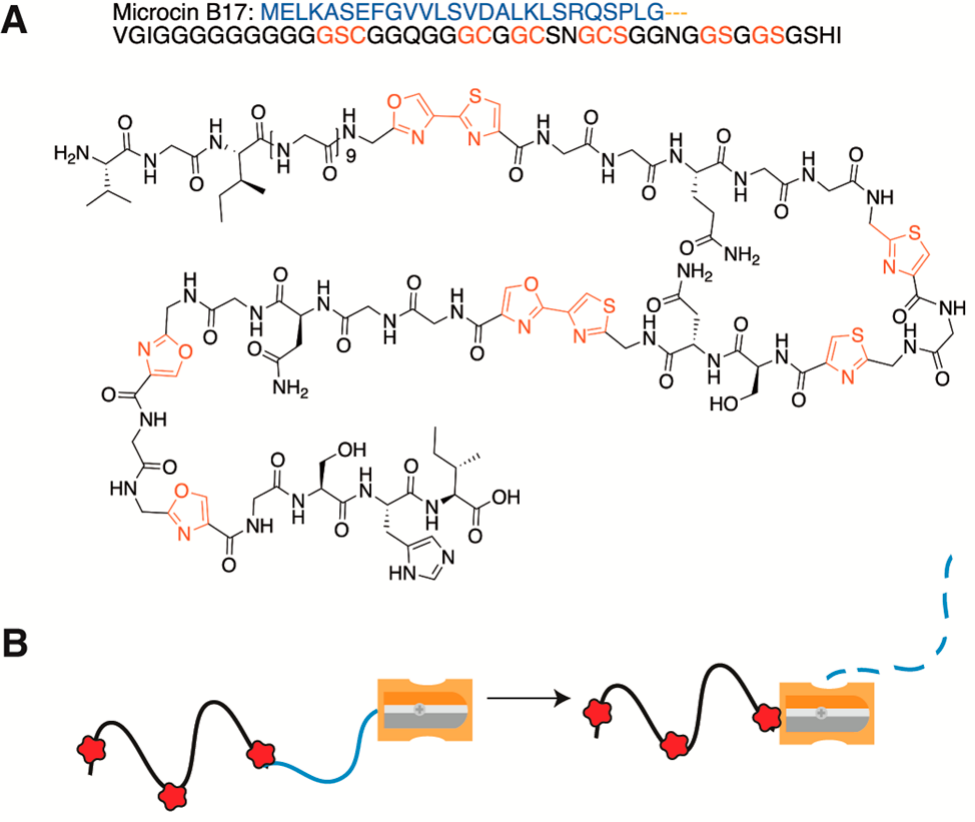
Structure of microcin B17 (A) and the schematic representation of the “pencil sharpener” mechanism of TldD/E (B). The LP is shown in blue and modifications in the CP are shown in red.

The ubiquitous and conserved heterodimeric metalloprotease TldD/TldE is encoded within the genomes of several RiPP producers, often outside of their BGCs, and has been implicated in their maturation. These proteins are present in ∼60% of all bacterial genomes, and genetic screens showed that production of the LAP microcin B17 in *Escherichia coli* relies upon TldD/E activity for LP removal (Figure 6A).^131,132^ Crystallization of the protease complex revealed TldD as the only metal binder using a conserved HExxxH motif, with TldE required for active complex formation. TldE has been suggested to have evolved from TldD (18% sequence identity) with loss of its catalytic residues; coevolution of TldD/E is supported by the increased stability of the heterocomplex compared to the respective homodimers.^133^ The TldD/E structure displayed a novel fold and resulted in a new classification in the MEROPS database (M103). The N-terminus of the microcin B17 precursor (McbA) appears to be threaded through the protease toward the active site where successive cleavage events occur, likened to the action of a pencil sharpener (Figure 6B).^133^ Proteolysis proceeds until the heterocycles sterically prevent further entry into the channel, resulting in cessation of proteolysis of the 13 residues N-terminal to the first heterocycle (Figure 6). In other words, the PTMs provide a ruler for the final proteolytic event. Consistent with this model, TldD/E was determined to be fairly substrate tolerant with respect to the LP, but a modified CP was required, suggesting that the unmodified peptide has a specific structure that prevents threading it into the protease channel. Substrate recognition occurs in a sequence-independent manner through two β-sheet interactions with TldD that clamp the peptide in the channel, with the side chains of the substrate pointing away from the protease, explaining the substrate tolerance. Modeling of a linear peptide bound to the protease suggests that a minimum of 15 amino acids is required at the N-terminus to access the active site for cleavage between residues 3 and 4 (release of a tripeptide), consistent with the linear N-terminal sequence of microcin B17 (Figure 6A). However, the narrow opening into the active site places stringency in processing only unfolded peptides.^133^ TldD/E-type proteases are also involved in LP removal of the RiPPs klebsazolicin in *Klebsiella pneumoniae* and a microcin C analogue in *Yersinia pseudotuberculosis* (McC^Yps^, Figures 5B and 5C).^134^ Once again, the cognate proteases are not encoded in the BGCs of these compounds. Consistent with this observation, TldD/E proteases are not only involved in the production of RiPPs but are believed to often have multiple other cellular functions.^135^ As a clear demonstration of the non-sequence-dependent substrate recognition, the TldD/E involved in microcin B17 biosynthesis was also able to remove the leader peptides from klebsazolicin and McC^Yps^ despite very diverse sequences.^134,136^

The currently characterized BGCs of the pearlin RiPP class encode either TldD/E-like or M50 family proteases.^137,138^ Pearlins are unusual among RiPPs in that the final mature compound is usually just a single, heavily modified amino acid that is made on a scaffold peptide that serves a similar role as a canonical LP. Hence, the scaffold-peptide-removing enzymes are technically carboxypeptidases. In addition, pearlin biosynthesis reuses the scaffold peptide to make multiple pearlins,^137^ and hence its cleavage needs to take place intracellularly. The activity of a TldD/E homologue encoded in the ammosamide BGC has yet to be confirmed,^138^ but if this enzyme indeed removes the scaffold peptide it cannot function similarly to the TldD/E involved in microcin B17 biosynthesis since that would successively cleave the scaffold peptide into many pieces.

The membrane-bound M50 proteases TglG and TmoG were shown to cleave 3-thia-amino acids from the C-terminus of their respective substrate peptides.^139,140^ TglG was intolerant toward C-terminal extension of the peptide substrate, but it was active with substrate peptides ending in both Glu and 3-thia-Glu; meanwhile, TmoG showed activity toward the TmoA peptide ending in either methionine or 3-thia-homoleucine.^139,140^

Investigations into the intramembrane M50 metalloprotease EpeP demonstrated the enzyme to be sufficient for production of the antimicrobial epipeptide EpeX*.^141^ EpeP is thought to be responsible for both the cleavage and export of the natural product. However, deletion of the *epeP* gene did not completely abolish EpeX* production, highlighting as yet unknown proteolytic and export systems in the native *Bacillus subtilis* producer.^141^

Bottromycin is an unusual RiPP as it does not have a canonical LP but instead a follower peptide that is attached to the CP.^142^ Its removal is performed by the dinuclear zinc-dependent amidohydrolase PurAH (an orthologue of BotAH).^143^ Macroamidine formation was shown to precede PurAH activity, and removal of the follower peptide makes macrocycle formation irreversible. Although bottromycin does not have a canonical leader peptide, the N-terminal Met residue still needs to be removed from the final product and a dedicated enzyme BotP is encoded in the BGC. BotP cleaves the N-terminal methionine of the precursor peptide BotA, congruent with other M17 leucine aminopeptidases.^144^ Crystallization and in vitro cleavage studies of the M17 family protease BotP investigated its substrate tolerance.^145^ Moreover, modeling studies suggested the importance of the sequence MGPV in the binding of BotP, and reduced in vitro activity of synthetic peptides in which Met was replaced by Leu or Ile supports the preference for an unbranched residue at the P1 position. Replacement of the native Gly in the P1′ position demonstrated some substrate tolerance as Ala, Ser, and Ile were tolerated, but efficiency was reduced when bulkier amino acids were introduced.^145^

## Two-Step Proteolytic Processing by Different Protease Family Enzymes

The biosynthesis of several RiPPs requires an additional proteolytic step after most of the LP is removed by a PCAT. Intriguingly, in almost all characterized examples involving lanthipeptides, the second proteolytic step removes a hexapeptide. Herein, we cover several examples of two-step proteolytic cleavage within class II lanthipeptides as well as the recently elucidated multistep maturation of autoinducing peptides.

**Figure 7:**
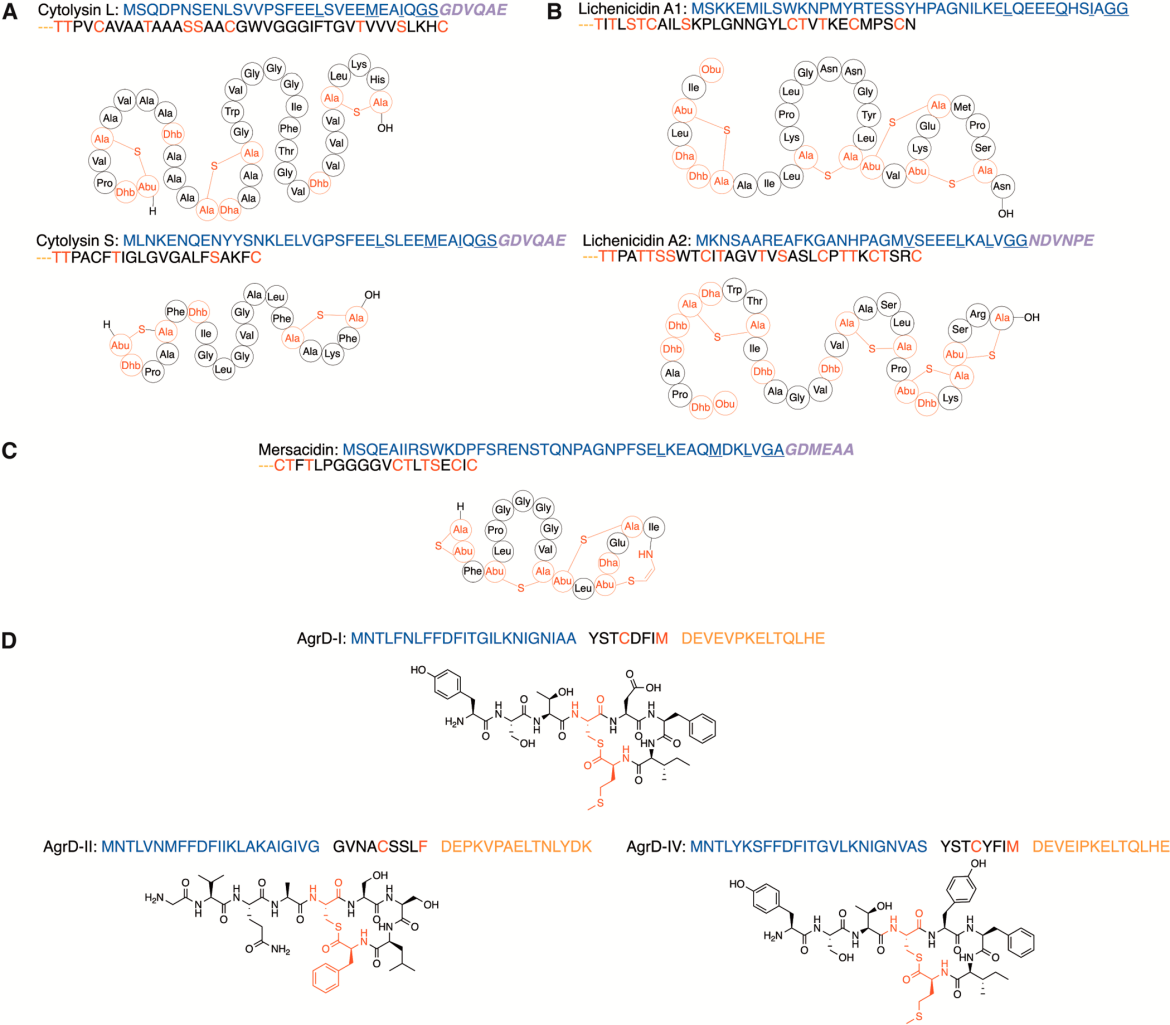
Representative examples of two-step removal of leader or follower peptides for (A) cytolysin, (B) lichenicidin, (C) mersacidin, and (D) AIP. The LP is shown in blue, and modifications in the CP are shown in red. Secondary cleavage sites are shown in purple and bolded italic font, whereas cleavage sites of follower peptides are shown in orange.

The two modified precursor peptides of the enterococcal two-component lanthipeptide cytolysin are first cleaved after a GS motif and exported by the PCAT CylB (Figure 7A).^42^ The extracellular serine protease CylA then acts on the two secreted products of CylB. Expressed as a preproenzyme, CylA undergoes signal peptide directed secretion followed by autocatalytic self-cleavage to yield the active form of the protease.^146^ Activated CylA then cleaves the remaining N-terminal hexapeptide GDVQAE from the two CylB products to yield the two components of bioactive cytolysin (cytolysin S and L, Figure 7A).^41^ CylA was also active against unmodified synthetic analogues of the precursor peptides consisting of a portion of the LP bearing the CylA cleavage site as well as the first two residues of the core peptide, demonstrating that specificity is not dependent on the PTMs.^41^ Furthermore, in vitro CylA was able to not only remove the hexapeptide but also the entire LP.^147^

The class II lanthipeptide lichenicidin constitutes another example of such a two-step processing (Figure 7B). The PCAT LicT and serine protease LicP operate similarly to CylB and CylA, respectively. First, an initial cleavage is performed by LicT after the GG motif of both modified precursor peptides, followed by removal of the remaining hexapeptide NDVNPE from the CP of one of the peptides by the serine protease LicP.^147,148^ Like CylA, LicP is made as a preproenzyme with a secretion signal peptide that undergoes a self-cleavage event to generate its active form, which was characterized crystallographically. As with cytolysin, in vitro studies established complete removal of the LP when the modified peptide was incubated with the serine protease alone, demonstrating that initial removal of most of the LP by LicT is unnecessary for in vitro reconstitution of LicP activity. Similarly, since LicT can process the LicA1 peptide that does not have the NDVNPE sequence (Figure 7B), it is not clear what the function is of the two-step LP removal process. LicP has tolerance for a variety of amino acids in the P1′ position, including Ser, Thr, Cys, Ile, and Gly, and although the enzyme prefers cyclized cognate substrates, it also cleaves linear substrates.^148^ With the observed substrate tolerance of the enzyme, biotechnological applications have been demonstrated such as traceless LP removal from unrelated systems or removal of affinity purification or solubilization tags from proteins by insertion of the recognition motif.^148,149^ A closely related enzyme is CerP involved in the biosynthesis of the cericidins, which also involves a two-step removal of the LP (Table 1).^150^ The β-peptides of the class II two-component lanthipeptides haloduracin,^43^ staphylococcin C55,^151^ and plantaracin W^152^ also undergo a two-step cleavage of the LP involving a second step that removes a hexapeptide, but the enzymes involved have not been characterized.

Similarly, for the class II lanthipeptide mersacidin, the PCAT MrsT first cleaves the LP of modified MrsA after the canonical GA motif, secreting inactive pre-MrsA (Figure 7C).^44^ The presence of the GDMEAA sequence after the cleavage site was necessary for MrsT activity.^45^ Further mutational studies indicated that removal of the Gly in the P1′ position to yield the sequence DMEAA prevented MrsT cleavage next to the resulting Asp residue.^45^ Premersacidin is then fully processed into its active form via cleavage of the N-terminal GDMEAA sequence by the extracellular protease AprE (a subtilisin protease, Table 1) in *Bacillus amyloliquefaciens*.^153^ This second cleavage step is necessary for successful proteolytic processing next to the N-terminal two-amino-acid lanthionine ring in mersacidin as MrsT (unlike LicT) was incapable of such activity; interestingly, the structurally similar lacticin 3147 (Figure 2A) is completely processed by a single PCAT LtnT, thereby highlighting the difference of MrsT as compared to its homologue.^45,154^

An unusual example of multistep proteolysis was discovered in the maturation of anti-inflammatory lanthipeptides from *Myxococcus fulvus* (Mfu). Genome mining revealed the presence of two proteases encoded in a class II lanthipeptide BGC, a PCAT and a M61 metalloprotease MfuP.^46^ The PCAT protease domain (MfuT150) was demonstrated to remove the LP at the double Gly motif. However, unlike other characterized M61 family members that are aminopeptidases, the M61 endopeptidase MfuP hydrolyzed the modified core peptide of MfuA within a lanthionine-containing macrocycle. Hence, this protease is not involved in LP removal, but appears to be used for CP modification. The function of this proteolytic event is not currently completely understood. Crystallization of MfuP confirmed the presence of a Zn-binding site, with activity abolished after EDTA treatment of the enzyme. Furthermore, MfuP was specific for the fully modified, pentacyclic MfuA core peptide compared to the tetracyclic mutants. This two-step proteolysis represents the first instance of combined PCAT and metalloprotease processing in the generation of a class II lanthipeptide.

A different type of combination of cysteine protease and metalloprotease activities was recently identified in the *Staphylococcus aureus agr* quorum sensing pathway. Pathway recapitulation of *agr* autoinducing peptide (AIP) biosynthesis had, until recently, remained elusive. The first step in AIP maturation involves thiolactone formation and concomitant cleavage of the C-terminal 14-mer from the AgrD peptide (e.g., Figure 7D) by the cysteine protease AgrB (C75 family). As an integral membrane protease, in vitro reconstitution of AgrB was only possible when embedded in a lipid membrane.^155^ The second step of AIP biosynthesis requires cleavage of the LP by another integral membrane metalloprotease (M79) MroQ. The necessity of MroQ in AIP production was demonstrated via generation of an *mroQ* knockout; deletion of the protease eliminated or severely reduced group I and II AIP production, which was restored with *mroQ* complementation.^156^ In vitro studies further showed that substrate specificity of MroQ is dependent on the linker peptide connecting the N-terminal α-helix and the C-terminal thiolactone of its substrate; AIPs containing the helix and C-terminal macrocycle of one AIP group and the linker of another AIP group were efficiently processed.^156^ However, whereas MroQ was shown to be active with groups I and II, and by homology group IV, AIP substrates, maturation of group III AIPs remains unresolved.

## Additional Protease Families

Hydrolase activities were confirmed in the biosynthesis of both crocagin and mycofactocin. The crocagin protease CgnD was annotated as a Ser esterase and belongs to the SGNH/GDSL hydrolase superfamily. In vitro data showed that the enzyme cleaved off the LP from a post-translationally modified intermediate with slow turnover rates.^157^ Since proteolysis occurs intracellularly, low activity may prevent premature cleavage of the precursor peptide. Meanwhile, the peptidase MftE, a creatininase homologue, catalyzes LP cleavage only after decarboxylation of the mycofactocin CP.^158^

## Nonprotease Peptide Backbone Cleavage Reactions during RiPP Biosynthesis

**Figure 8:**
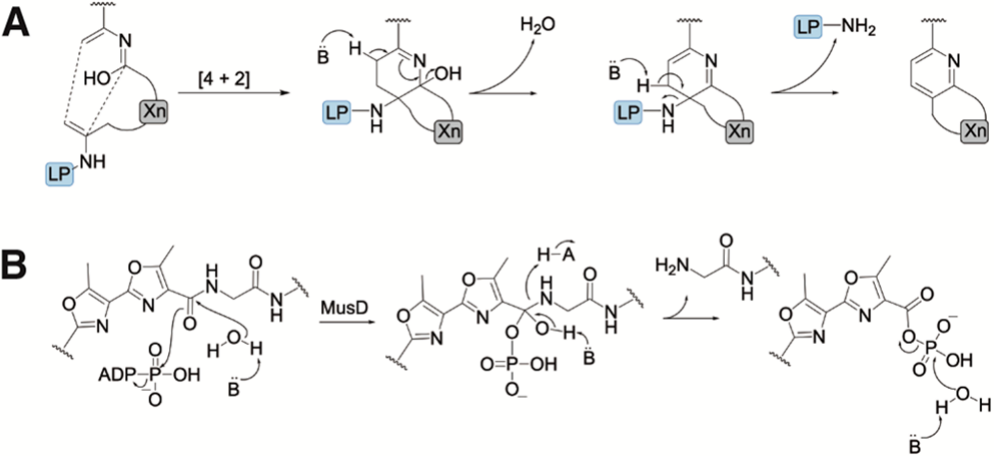
Schematic for peptide backbone cleavage during (A) [4 + 2] heterocyclization and (B) the proposed mechanism of biosynthesis of cyanobactin muscoride D.

We have discussed protease families involved in RiPP maturation, but it is of note that natural products belonging to pyritides and thiopeptides utilize proteins that perform heterocyclization reactions during which the LP is removed (Figure 8A).^159,160^ Recently, another mechanism of peptide bond cleavage that does not require a protease was reported for the YcaO-like ATP-dependent enzyme MusD involved in the formation of the linear cyanobactin muscoride A. A cryo-EM structure of MusD and in vitro reconstitution of its activity revealed ATP-mediated hydrolysis of the terminal GV residues; during this process, H_2_O acts as a nucleophile that reacts with an acyl phosphate intermediate (Figure 8B).^161^ Thus, MusD removes a C-terminal recognition sequence by phosphorolysis followed by hydrolysis as opposed to proteolysis by the PatG C-terminal to the CP that leads to cyclic cyanobactins (Figure 4). MusA substrates bearing either bis-azole or two thiazoles were accepted by MusD, indicating a level of chemo-tolerance.^161^

## Summary and Outlook

Protease-dependent maturation of precursor peptides remains a relatively unexplored area within studies of RiPP biosynthesis. Surprisingly diverse mechanisms have been uncovered for leader peptide removal including single- or multistep proteolysis. Additionally, an array of protease specificities have been reported ranging from highly specific for the modified RiPP (e.g., FlaP) to broad substrate tolerance with a length dependence (e.g., TldD/E). The Cys proteases LahT150 and PoyH have great utility for the removal of double Gly type leader peptides but are not universally active. For example, LahT150 is not able to remove LPs that do not have the three hydrophobic amino acids at positions −4, – 7, and −12.^28,75^ Presumably, among the many PCATs in the genomes, there may be other protease domains that have activity as stand-alone peptidases that may expand the catalogue of broad-substrate enzymes that can be used for the removal of LPs with a double glycine motif. Similarly, the serine proteases LicP and CylA have been used for sequence-specific, traceless peptide bond cleavage by insertion of their recognition motifs into noncognate peptides and proteins, and AplP-like proteases have demonstrated broad utility for class III lanthipeptides.

There are ongoing questions regarding RiPP classes with putative proteases for which activity has not been confirmed in vivo or in vitro (Table 1). Furthermore, the generation of a number of RiPP natural products by their native producers is dependent upon as yet unidentified host proteases that are typically not encoded in the BGCs (Table 1). The recent correlation workflow established for lanthipeptides^130^ may allow identification of proteases for these other RiPP families. Finally, the mechanisms underpinning substrate recognition by the proteases of many RiPP classes have yet to be elucidated. Continued research in these areas will likely afford the natural product community greater ease of production and diversification of compounds with various bioactivities.
